# Identification of the nicotinamide mononucleotide adenylyltransferase of *Trypanosoma cruzi*

**DOI:** 10.1590/0074-02760150175

**Published:** 2015-11

**Authors:** Carlos H Niño, Nicolás Forero-Baena, Luis E Contreras, Diana Sánchez-Lancheros, Katherine Figarella, María H Ramírez

**Affiliations:** 1 Universidad Nacional de Colombia, Facultad de Ciencias, Laboratorio de Investigaciones Básicas en Bioquímica, Bogotá, Colombia; 2 Fundación Instituto de Estudios Avanzados, Caracas, Venezuela

**Keywords:** nicotinamide adenine dinucleotide, nicotinic acid mononucleotide, nicotinamide mononucleotide, nicotinamide mononucleotide adenylyltransferase, *Trypanosoma cruzi*, Chagas disease

## Abstract

The intracellular parasite *Trypanosoma* cruzi is the aetiological agent of Chagas disease, a public health concern with an increasing incidence rate. This increase is due, among other reasons, to the parasite's drug resistance mechanisms, which require nicotinamide adenine dinucleotide (NAD^+^). Furthermore, this molecule is involved in metabolic and intracellular signalling processes necessary for the survival of *T. cruzi* throughout its life cycle. NAD^+^ biosynthesis is performed by *de novo* and salvage pathways, which converge on the step that is catalysed by the enzyme nicotinamide mononucleotide adenylyltransferase (NMNAT) (enzyme commission *number*: 2.7.7.1). The identification of the NMNAT of *T. cruzi* is important for the development of future therapeutic strategies to treat Chagas disease. In this study, a hypothetical open reading frame (ORF) for NMNAT was identified in the genome of *T. cruzi*. The corresponding putative protein was analysed by simulating structural models. The ORF was amplified from genomic DNA by polymerase chain reaction and was further used for the construction of a corresponding recombinant expression vector. The expressed recombinant protein was partially purified and its activity was evaluated using enzymatic assays. These results comprise the first identification of an NMNAT in *T. cruzi* using bioinformatics and experimental tools and hence represent the first step to understanding NAD^+^ metabolism in these parasites.

Nicotinamide adenine dinucleotide (NAD^+^) and its phosphorylated form (NADP^+^) are vital coenzymes for cellular physiology in all organisms. These dinucleotides perform important functions in cellular basal metabolism and cellular antioxidant mechanisms (Kirsch & De Groot 2001, Ghosh et al. 2010). NAD^+^ has an equally important function as a substrate and is involved in multiple cellular signalling processes (Berger et al. 2004). Because of this role, NAD^+^ is continuously consumed and thus, its synthesis is essential to maintain cellular homeostasis. NAD^+ ^synthesis occurs *via* two pathways: *de novo* synthesis and salvage pathways. In both cases, nicotinic acid mononucleotide (NAMN) and nicotinamide mononucleotide (NMN) are synthesised, with the subsequent synthesis of NAD^+^ from these precursors. In the final stage, both synthetic pathways must converge on the reaction catalysed by NMN adenylyltransferase (NMNAT) [enzyme commission number (EC): 2.7.7.1], which transfers the adenylate from adenosine triphosphate (ATP) to any of the mononucleotides (NMN or NAMN), producing NAD^+^ or nicotinic acid adenine dinucleotide (NAAD) along with pyrophosphate (Jayaram et al. 2011). NMNAT has been identified in archaebacteria, eubacteria and eukaryotes. Nonetheless, despite their ubiquity, large differences exist between these enzymes in terms of substrate specificity, oligomerisation capabilities and sensitivity to divalent cationic cofactors (Lau et al. 2009).

*Trypanosoma cruzi *is a trypanosomatid that causes Chagas disease, an important public health concern in Latin American countries given its high prevalence and its high morbidity and mortality rates (Rassi Jr et al. 2012). The increase in diseases caused by trypanosomatids is largely due to the development of resistance to commonly used drugs against these parasites (Borges et al. 2005). Redox systems are fundamental for the survival and drug resistance of intracellular parasites (Krauth-Siegel et al. 2003, Müller et al. 2003). All known resistance mechanisms require NAD^+^ for their operation (Müller et al. 2003, Turrens 2004). Moreover, NAD(P) biosynthetic pathways can be considered a generous source of enzymatic targets for drug development (Magni et al. 2009). The study of NAD^+^ metabolism in *T. cruzi* would allow for the design of strategies for the treatment of Chagas disease given the importance of this nucleotide. Consequently, the identification of NMNAT enzymes in this parasite would represent an increase in knowledge of NAD^+^synthesis and aid in the search for new drug targets.

This paper presents, for the first time, the *in silico *identification of an NMNAT of *T. cruzi* (TcNMNAT) using cloning and the heterologous expression of a candidate gene to confirm its identity using direct enzymatic assays.

## MATERIALS AND METHODS

*Bioinformatic identification of TcNMNAT* - Initially, to obtain candidate sequences, a multiple alignment was performed of 16 amino acid (aa) sequences corresponding to several NMNATs of various organisms with varying degrees of phylogenetic divergence. Such sequences were indexed and manually annotated in the UniProtKB protein database (UniProt Consortium 2014). To perform the alignment, the Multiple Sequence Comparison by Log-Expectation algorithm (Edgar 2004) embedded as a plugin in the software CLC Sequence Viewer v.6.9 (CLC bio*,*Denmark) was used. The resulting consensus sequence was used to search the genomes of the available *T. cruzi *strains using the BLASTP algorithm in the trypanosomatid TriTrypDB database (Aslett et al. 2010).

*Bioinformatic analysis of the TcNMNAT putative protein structure* - The primary, secondary and tertiary structures of the TcNMNAT protein encoded in the*T. cruzi *CL Brener Esmeraldo-like genome were analysed. The web-based tool ProtParam from the ExPASy server (Gasteiger et al. 2003) was used for the analysis of the primary structure and provided predictions of different physicochemical aspects of the protein, such as length and molecular mass. The secondary structure patterns were predicted using three different algorithms that were located on the web-based server NPS@ for network protein sequence analysis (Combet et al. 2000): the GORIV algorithm (Garnier et al. 1996), the third-generation algorithm PHD (Rost & Sander 1993) and the PREDATOR algorithm (Frishman & Argos 1996). For the prediction of the tertiary structure and for the assignment of a hypothetical function, a tridimensional model of the putative sequence was performed using the I-TASSER server (Zhang 2008, Roy et al. 2010, 2012), 2012). The parameters that were chosen from this server were the best tridimensional model that was generated, the prediction of the Enzyme Commission Number EC number and the gene ontology (GO) terms. The tridimensional model that was generated was used for a structural alignment with the HsNMNAT-1 model (*PDB ID:* 1KQO) (Zhou et al. 2002), which was generated from the X-Ray Diffraction (XRD) database and registered on the PDB database (Bernstein et al. 1978). This process was performed using UCSF Chimera v.1.8 software (Pettersen et al. 2004).

*T. cruzi culture and DNA extraction* - *T. cruzi*epimastigotes were cultured at 27ºC in Schneider medium (Sigma S9895) that was supplemented with 10% (v/v) foetal bovine serum (Gibco) (Baker & Price 1973, Miralles et al. 2002). DNA was extracted from 1 x 10^7^ epimastigotes using the phenol chloroform method (Pereira et al. 2000).

*Amplification of the coding fragment of TcNMNAT* - The amplification was performed by polymerase chain reaction (PCR) using the primers 5'-CACCATGAGCGATGACACA-3´ and 5'-TCAACAATTTTGAGTATTGTTTG-3' (Dieffenbach et al. 1993). Amplification was performed using two systems. The fragment's initial amplification was achieved with a Platinum^®^ PCR SuperMix High Fidelity (Invitrogen) system using 200 ng of genomic DNA as a template. An initial denaturation step of 5 min at 94ºC was used, followed by 30 cycles of denaturation at 94ºC for 30 s, annealing at 50ºC for 30 s and extension at 72ºC for 1 min. The final extension step was 10 min at 72ºC. A second amplification was subsequently performed using 1 μL of the previous PCR product as template; in this case, a Pfu DNA Polymerase system (Fermentas, USA) was used. The initial denaturation was 5 min at 95ºC, followed by 30 cycles of denaturation at 95ºC for 30 s, annealing at 50ºC for 30 s and extension at 72ºC for 2 min. The final extension step was 10 min at 72ºC.

*Cloning and expression of TcNMNAT* - Using the amplified fragment and the pET100 D-TOPO Vector (Invitrogen), the construct pET100 D-TOPO-TcNMNAT was designed. The insertion of the PCR fragment was verified using digestion with the restriction enzyme EcoRV (Fermentas). Using this construct, *Escherichia coli* BL21 (DE3) cells were transformed by thermal shock. The transformed clones were inoculated into liquid Luria broth (LB) that was supplemented with 100 μg/mL ampicillin and incubated overnight at 37ºC. The cultures were diluted (1:50) in the same medium. When the cultures reached an optical density at 600_nm_ of 0.6, the recombinant protein was induced overnight at 37ºC using isopropyl β-D-1-thiogalactopyranoside (IPTG) at a final concentration of 1 mM. The recombinant protein was expressed with a histidine tag (6xHis) on its N-terminus to allow for its detection and further purification. The expression of the recombinant protein was confirmed using sodium dodecyl sulfate polyacrylamide gel electrophoresis (SDS-PAGE) and western blotting (WB) (Fra et al. 1995, Ramprasad et al. 1996)*.*

*Purification of 6xHis-TcNMNAT* - The induced BL21 (DE3) cell cultures were centrifuged at 4,000 *g *for 15 min at 4ºC and resuspended in lysis buffer (300 mM NaCl; 50 mM NaH_2_PO_4_; 10 mM imidazole, pH 8.0/NaOH). Lysozyme (1 mg/mL) and protease inhibitors (P8340; Sigma) were then added. The mixture was incubated for 30 min at 4ºC with constant agitation. After incubation, the sample was macerated with liquid nitrogen and centrifuged at 19,000*g* for 15 min at 4ºC. The soluble protein fraction (supernatant) was collected. The obtained soluble extracts were partially purified using nickel affinity chromatography with a staggered elution scheme. For this scheme, a nickel resin with nitrilotriacetic acid (Ni-NTA) was used as the stationary phase. As the mobile phase, lysis buffer (50 mM NaH_2_PO_4_, 300 mM NaCl pH 8.0/NaOH) was used with varying imidazole concentrations according to both the manufacturer's instructions (Qiagen) and the purification step. The resin was equilibrated with lysis buffer with 10 mM imidazole before the protein lysate was added and the same 10 mM imidazole concentration was used for the washes. Finally, several consecutive elutions were performed with increasing imidazole concentrations in lysis buffer as follows: first, three elutions with 50 mM imidazole were performed; then, two elutions with 75 mM imidazole and, finally, four elutions with 250 mM imidazole. The proteins that did not bind to the resin, the eluates and the washes were analysed by SDS-PAGE and WB (Fra et al. 1995, Ramprasad et al. 1996).

*Detection of recombinant proteins using WB* - The samples were separated by SDS-PAGE and then transferred to a polyvinylidene fluoride (Thermo-Scientific) membrane using a 200 mA current for 2 h in transfer buffer [0.2 M glycine, 10 mM Tris/HCl pH 8.0, 10% (v/v) methanol]. The membrane was blocked overnight with a 5% (w/v) nonfat milk solution in tris-buffered saline with Tween 20 (TBS-T) [150 mM NaCl, 20 mM Tris/HCl pH 7.0, 0.1% (v/v) Tween-20]. The recombinant protein was detected using the primary antibody anti-6xHis (1:3,000), the secondary antibody biotinylated anti-mouse IgG (1:2,000) and streptavidin-alkaline phosphatase (1:3,000) in TBS-T for the final detection with nitro blue tetrazolium (S380C; Promega) and 5-bromo-4-chloro-3-indolyl-phosphate (S381C; Promega) substrates (Ramprasad et al. 1996).

*Direct enzymatic assays* - The enzymatic activity of the partially purified recombinant proteins was determined by direct enzymatic assays. The reaction mix contained 25 mM HEPES/KOH pH 7.4, 10 mM MgCl_2_, 1 mM ATP (Sigma) and 1 mM NMN (Sigma) or 1 mM NAMN (Sigma). This mix was incubated at 37ºC for 5 min and the enzymatic reaction was initiated by the addition of 5 μg of the samples: the partially purified protein 6xHis-TcNMNAT and the recombinant form of the human isozyme 6xHis-HsNMNAT3 that was expressed from the vector pQE-30/hsnmnat3 as a positive control. The assays were performed in 100 μL at 37ºC for 30 min. To stop and neutralise the reactions, 1.2 M HClO_4_ and 1 M K_2_CO_3_ were used, respectively (Emanuelli et al. 1999). The analyte separation (50 μL) was performed by reverse phase high-performance liquid chromatographic (RP-HPLC) using a C18 column, 25 cm long x 4.6 mm internal diameter, with a particle size of 5 μm (Phenomenex). An elution gradient was used with phosphate buffer (0.1 mM potassium phosphate, pH 6.0) and methanol-phosphate buffer [0.1 mM potassium phosphate pH 6.0 and 20% (v/v) methanol]. The separations were performed at room temperature with a flow of 1.5 mL/min. The analyte detection was performed at 254 nm, considering the elution area under the peak and compared with the appropriate pattern (NAD^+^ or NAAD).

*Protein quantification* - The protein concentration was determined by densitometry analysis and/or the Bradford method using bovine serum albumin (BSA) as a standard (Bradford 1976).

## RESULTS

**Fig. 1:**
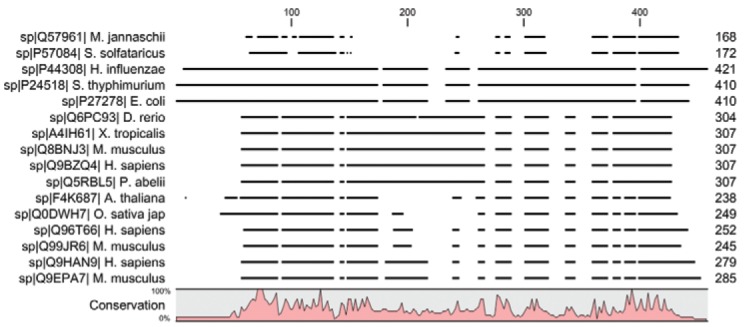
multiple alignments of 16 nicotinamide mononucleotide adenylyltransferase isozymes from phylogenetically divergent organisms. Sequences used were indexed and annotated in the UniProtKB database and are shown with their corresponding identification code and the degree of conservation of the residues along the sequences.

*Identification of NMNAT candidate sequences in the genome of T. cruzi* - Based on a multiple sequence alignment of NMNAT isozymes (Fig. 1), a consensus sequence of 244 residues was obtained. This sequence was composed of residues that were highly conserved among the initial sequences. Using this sequence, a search was performed on the genomes of four *T. cruzi *strains using the BLASTP algorithm. A candidate sequence was found for each genome, producing a total of four sequences with scores and significant p-values as shown in Table I; each of these genes encodes a protein of 289 aa and 32 kDa from a coding region of 870 nucleotides. For all four genes, a biosynthetic function (predicted GO process) and a nucleotidyltransferase activity were predicted (predicted GO function).

**TABLE I:** BLASTP search results for NMNAT in genomes of *Trypanosoma cruzi *strains

Gen	Score	p (n)	Gene ID	Organism	Genomic location	Predicted gene ontology function
1	238	5.3E-021	TcCLB.507047.170	*T. cruzi* CL Brener Esmeraldo-like	TcChr17-S: 188,592-189,461 (-)	Nucleotidyltransferase activity
2	237	6.8E-021	TCSYLVIO_008891	*T. cruzi* Sylvio X10/1	ADWP02020923: 12,533-13,402 (-)	Nucleotidyltransferase activity
3	232	2.3E-020	Tc_MARK_7577	*T. cruzi* marinkellei strain B7	TcMARK_CONTIG_420: 29,693-30,562 (-)	Nucleotidyltransferase activity
4	227	7.8E-020	TcCLB.509179.80	*T. cruzi* CL Brener non-Esmeraldo-like	TcChr17-P: 188,641-189,510 (-)	Nucleotidyltransferase activity

*Bioinformatics analysis of the putative TcNMNAT* - Using the sequence from the genome of CL Brener Esmeraldo-like (TcCLB.507047.170), a structural analysis of the putative TcNMNAT was performed. The primary structure showed that the putative protein is 289 aa and has a molecular mass of 32,031.4 Da. The modelling of the secondary structure pattern was performed using three different algorithms (Table II). The GORIV algorithm (precision ~64%) is based on the propensity of each aa and its neighbours to form a determined secondary structure using a dynamic window of a determined length. The third-generation algorithm PDH (precision >70%) is based on adaptive neural networks and the PREDATOR algorithm (precision ~68-75%) is based on the recognition of aa that are potentially linked by hydrogen bonds. All three bioinformatics methods showed similar results, with a mean of 43.90% residues located in α-helices, 12.57% in β-sheets and 44.52% in random regions.

**TABLE II:**
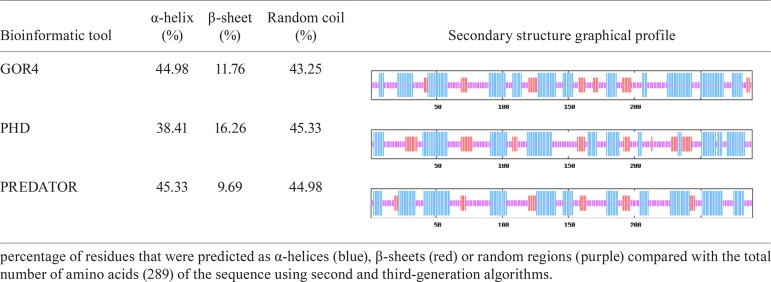
Secondary structure prediction for a nicotinamide mononucleotide adenylyltransferase of *Trypanosoma cruzi* candidate

**Fig. 2A:**
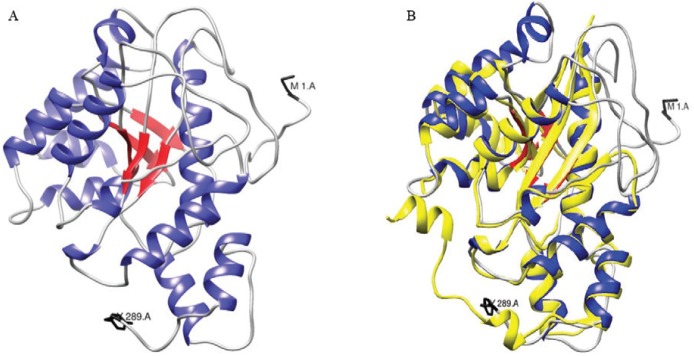
tertiary structure model of the nicotinamide mononucleotide adenylyltransferase of *Trypanosoma cruzi* (TcNMNAT) hypothetical sequence using the I-TASSER server. α-helices are shown in blue and β-sheets are shown in red. The N-terminal methionine and C-terminal tyrosine residues are shown in black (C-score: -5 < -0.70 < 2, TM-score: 0.62 ± 0.14 > 0.5, RMSD: 7.6 ± 4.3 Å); B: overlap of the TcNMNAT model with the tertiary structure of the HsNMNAT (1KQO) protein. HsNMNAT is shown in yellow and TcNMNAT is shown in red and blue (RMSD between 170 atom pairs: 0.897 Å).

For the modelling of the tertiary structure and the assignation of a putative function, a tridimensional model was constructed for the sequence using the I-TASSER server. The first of five models that were constructed by the server which had the best quality parameters (RMSD, C-score and TM-score) is shown in Fig. 2A. This figure shows a Rossmann fold, which is typical of proteins that bind to nucleotides (βαβαβ motif). The three-dimensional model confirmed the secondary structure models that were predicted by the GORIV, PDH and PREDATOR algorithms. Fig. 2B shows a tridimensional alignment with the tertiary structure of the HsNMNAT (1KQO) as generated from XRD data. Clear structural coincidences were evident in several α-helices and β-sheets of the overlapping proteins, which had an RMSD of 0.897 Å between 170 atom pairs.

Additionally, the I-TASSER server predicted the function of the protein. The EC number, which was predicted from comparisons with protein structures that were registered in databases, was 2.7.7.1/18, which is consistent with an adenylyltransferase function of the NMNAT. Likewise, the predicted GO terms for the function of the protein are consistent with the functional characteristics that are known for NMNAT proteins: GO:0005524, ATP binding (94% confidence), GO:0004515, nicotinate-nucleotide adenylyltransferase activity (94% confidence) and GO:0000309, nicotinamide-nucleotide adenylyltransferase activity (75% confidence).

**Fig. 3:**
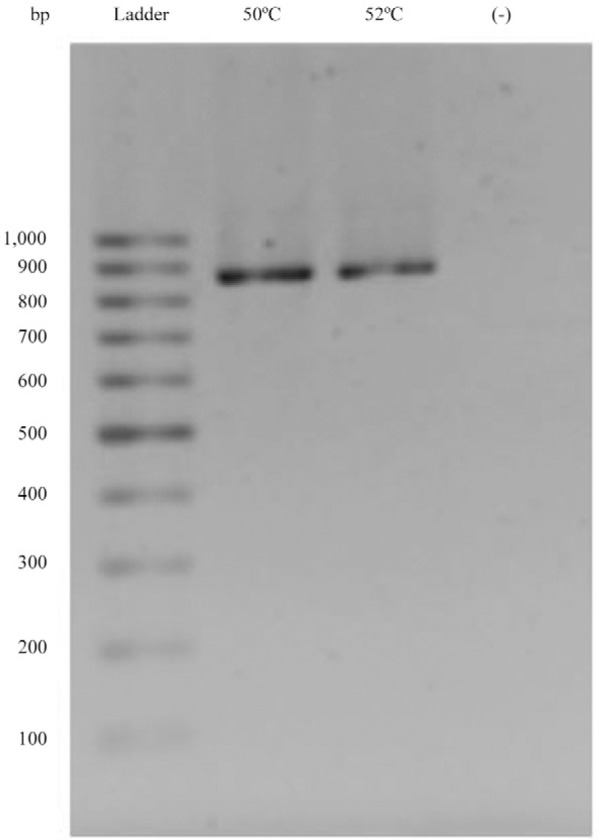
open reading frame amplification of nicotinamide mononucleotide adenylyltransferase of *Trypanosoma cruzi* from genomic DNA. Electrophoresis on a 1% agarose gel in Tris/borate/ethylenediamine tetraacetic acid of the amplified fragment. Lanes correspond to molecular marker (100 bp ladder), amplicons from genomic DNA using annealing temperatures of 50°C and 52ºC, followed by the negative control (polymerase chain reaction with no template). For subsequent experiments, a temperature of 50ºC was used.

*TcNMNAT cloning* - Taking into account the sequence of the*TcCLB.507047.170* gene from the genome of CL Brener Esmeraldo-like, primers for the subsequent PCR amplification were designed.*T. cruzi* strain CL Brener genomic DNA was used as a template. A single fragment with an approximate size of 870 bp was amplified according to the electrophoresis analysis (Fig. 3). Two annealing temperatures below the hypothetical value for the primers were used to observe which condition would render more specific amplification. This fragment was directly ligated into the expression vector pET100 D-TOPO, followed by transformation into the maintenance *E. coli *strain TOP10.

**Fig. 4:**
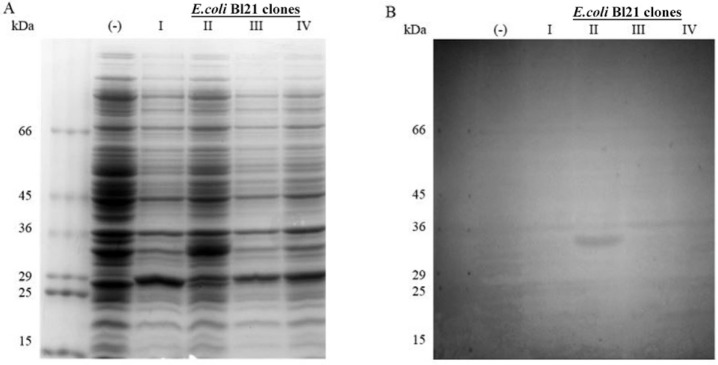
induction of the 6xHis-nicotinamide mononucleotide adenylyltransferase of *Trypanosoma cruzi* (TcNMNAT) in total cellular extracts from transformed BL21 DE3 cells. A: sodium dodecyl sulfate polyacrylamide gel electrophoresis on a 10% gel stained with Coomassie dye; B: western blot with alkaline phosphatase. The lanes include the molecular weight marker (kDa), induced negative control with a nontransformed strain (-) and different inductions of transformed cells with the expression vector pET100 D-TOPO-6xHis-TcNMNAT (BL21 clones I, II, III and IV).

*Expression and purification of 6xHis-TcNMNAT* - The cloned plasmids were extracted and used to transform *E. coli* BL21 DE3 cells. The expression of the recombinant protein, termed 6xHis-TcNMNAT, was induced at 37ºC overnight with 1 mM IPTG (Fermentas). Expression was monitored using SDS-PAGE and WB against the histidine tag that was added to the insert sequence during the cloning step in the pET-100 D-TOPO vector (Fig. 4). The SDS-PAGE in Fig. 4A shows that only one of the TcNMNAT-BL21 DE3 clones that were analysed overexpressed a 35-kDa band, which corresponded to the expected molecular weight for the original aa sequence (32 kDa) with the histidine tag added (3 kDa). The identity of this recombinant protein was confirmed using an anti-histidine antibody (Fig. 4B).

**Fig. 5:**
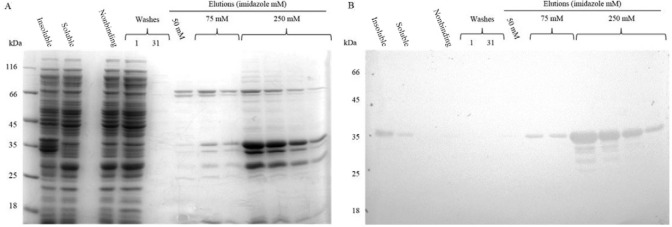
nickel affinity chromatography using Ni-NTA with staggered elutions. A: sodium dodecyl sulfate polyacrylamide gel electrophoresis on a 12% gel stained with Coomassie dye; B: western blot with alkaline phosphatase. The lanes correspond to the molecular weight marker, the insoluble fraction of induced *Escherichia coli* BL21 DE3 cells, the soluble fraction of induced *E. coli* BL21 DE3 cells, proteins that did not bind to the Ni-NTA resin, the first wash fraction (L1), the final wash fraction (L31), the third elution at 50 mM imidazole and elutions using 75 mM and 250 mM imidazole.

The recombinant protein was purified from the soluble fraction of the induced cells, although the majority of the 6xHis-TcNMNAT was found in the insoluble fraction. This process was performed by nickel affinity chromatography using Ni-NTA with a staggered elution scheme (using imidazole), as shown in Fig. 5. A partial purification and an enrichment of the recombinant protein were obtained. The bands with a lower molecular weight correspond to possible degradation products according to the recognition of the antibody.

**Fig. 6:**
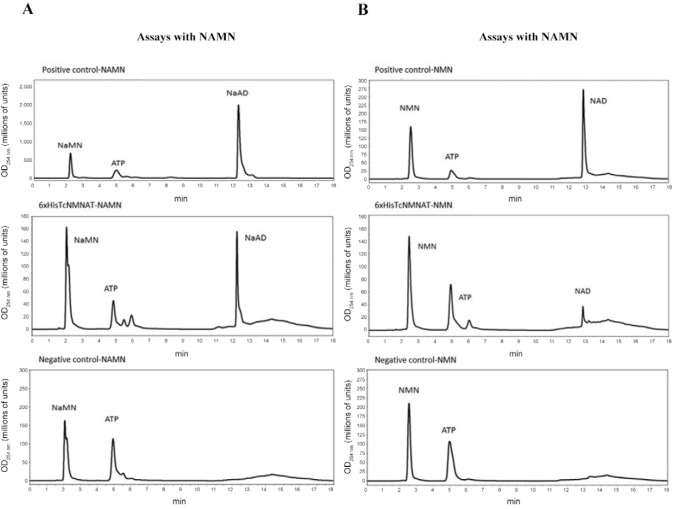
determination of the enzymatic activity of 6xHis-nicotinamide mononucleotide adenylyltransferase of *Trypanosoma cruzi*(TcNMNAT). A: assays performed with nicotinic acid mononucleotide (NAMN); B: assays performed with nicotinamide mononucleotide (NMN). From the top down, the graphs correspond to positive controls (6xHis-HsNMNAT3, the recombinant form of the human NMNAT isozyme), assays with the 6xHis-TcNMNAT and the negative controls (reactions with no enzyme); ATP: adenosine triphosphate; NAD: nicotinamide adenine dinucleotide; NaMN: nicotinic acid mononucleotide; OD: optical density.

*Confirmation of the NMNAT identity using enzymatic assays* - The functional identity of the recombinant protein was verified with direct enzymatic assays using the 250 mM imidazole elution fraction from the affinity chromatography. In these assays, the presence of substrates and products was detected using RP-HPLC (Fig. 6). The chromatographic profiles showed that the hypothetical protein 6xHis-TcNMNAT is an NMNAT, which was confirmed by the presence of the corresponding products. This enzyme showed activity for both NAMN and NMN. NAMN and NMN are the substrates that are used by NMNAT in the presence of ATP to form NAD^+^ and NAAD, respectively (Berger et al. 2004). Therefore, this result confirms that 6xHis-TcNMNAT effectively belongs to the NMNAT family.

## DISCUSSION

NAD^+ ^synthesising routes in *T. cruzi *have been previously reported, including the NMNAT gene (Klein et al. 2013). However, in the present study, a TcNMNAT was identified by means of different bioinformatic and experimental strategies. This finding represents important progress towards the understanding of the biosynthesis of pyridine nucleotides in parasitic protozoa. In addition, this study establishes a first approach to understanding NAD^+^ metabolism in *T. cruzi* by providing information about NAD^+^ synthesis.

The performed sequence analysis allowed for the identification of a single gene encoding NMNAT in the different genomes that are available for *T. cruzi*strains (Table I). The structural analysis of the putative protein, which is encoded by a gene from the strain CL Brener Esmeraldo-like, suggests that it belongs to the NMNAT family (Fig. 2). Based on this gene, a sequence was amplified from the genomic DNA of *T. cruzi *CL Brener (Fig. 3) and the approximately 35 kDa recombinant protein 6xHis-TcNMNAT was successfully expressed (Fig. 4). In addition, partial purification of the protein was achieved (Fig. 5). The purified recombinant protein was capable of synthesising NAD^+^ and NAAD from NAMN and NMN (Fig. 6), respectively, which are substrates that used by the NMNAT family of enzymes (Jayaram et al. 2011). These results indicate that the hypothetical sequence analysed*in silico *and in vitro corresponds to that of the TcNMNAT*.*

All of the known pathways leading to the synthesis of NAD^+^ require NMNAT. This enzyme is of great importance for metabolism, energy supply and signal transduction (Berger et al. 2004, Jayaram et al. 2011). This enzyme also plays an important role in oxidative stress control and drug resistance. Therefore, the TcNMNAT represents a potential target for the development of future drugs and other therapeutic strategies, as has been proposed in other intracellular parasites (Sorci et al. 2009).

As no other NMNAT structurally related enzymes were found, it is tempting to suggest that the NAD^+^ levels in this organism may depend exclusively on the expression of the NMNAT that was identified in this study. Likewise, the production of NAD^+^ could be explained by the use of precursors from the host. However, the existence of additional isozymes in *T. cruzi* should not be excluded. It is necessary to use other search strategies to identify new candidates that do not meet the current prediction parameters (e.g., gene finding through hidden Markov models).

This is the first NMNAT to be identified in the genus *Trypanosoma*, which includes several pathogens that infect humans and other economically important mammals. Thus, TcNMNAT emerges as a starting point in the understanding of the NAD^+ ^synthetic pathway in this parasite.
